# Timely meta-analysis on the efficacy of adoptive immunotherapy for hepatocellular carcinoma patients after curative therapy

**DOI:** 10.1371/journal.pone.0174222

**Published:** 2017-03-24

**Authors:** Han-Yue Mo, Ying-Yang Liao, Xue-Mei You, Alessandro Cucchetti, Bao-Hong Yuan, Ru-Hong Li, Jian-Hong Zhong, Le-Qun Li

**Affiliations:** 1 Department of Chemotherapy, Affiliated Tumor Hospital of Guangxi Medical University, Nanning, China; 2 Department of Nutrition, Affiliated Tumor Hospital of Guangxi Medical University, Nanning, China; 3 Department of Hepatobiliary Surgery, Affiliated Tumor Hospital of Guangxi Medical University, Nanning, China; 4 Guangxi Liver Cancer Diagnosis and Treatment Engineering and Technology Research Center, Nanning, China; 5 Department of Medical and Surgical Sciences, S. Orsola-Malpighi Hospital, Alma Mater Studiorum, University of Bologna, Bologna, Italy; 6 Department of General Surgery, Yan’An Hospital Affiliated to Kunming Medical University, Kunming, China; Taipei Veterans General Hospital, TAIWAN

## Abstract

**Aims:**

The role of adoptive immunotherapy (AIT) for patients with hepatocellular carcinoma (HCC) who have received curative therapy is still not well illustrated. This timely meta-analysis aims to update the current evidence on efficacy and safety of AIT for patients with HCC who have received curative therapy.

**Methods:**

We searched PubMed, EMBASE, Scopus and the Cochrane Library Through January 2017 for relevant studies. Mortality and tumor recurrence were compared between patients with or without adjuvant AIT. The meta-analysis was performed using Review Manager 5.3.

**Results:**

Eight studies involving 1861 patients met the eligibility criteria and were meta-analyzed. Adjuvant AIT was associated with significantly lower mortality at 1 year (RR 0.64, 95%CI 0.52–0.79), 3 years (RR 0.73, 95%CI 0.65–0.81) and 5 years (RR 0.86, 95%CI 0.79–0.94). Similarly, adjuvant AIT was associated with significantly lower recurrence rate than curative therapies alone at 1 year (RR 0.64, 95%CI 0.49–0.82), 3 years (RR 0.85, 95%CI 0.79–0.91) and 5 years (RR 0.90, 95%CI 0.85–0.95). Short-term outcomes were confirmed in sensitivity analyses based on randomized trials or choice of random- or fixed-effect meta-analysis model. None of the included patients experienced grade 4 adverse events.

**Conclusions:**

This timely meta-analysis confirms the evidence that adjuvant AIT for patients with HCC after curative treatment lowers risk of mortality and tumor recurrence.

## Introduction

Hepatocellular carcinoma (HCC) is a common cancer worldwide and ranking as the third most common cause of cancer mortality [1]. Moreover, only a small proportion of patients with HCC are with early stage HCC [2]. Hepatic resection, radiofrequency ablation, and percutaneous ethanol injection are curative treatments for such patients [3]. However, 5-year median disease-free survival after these treatments is only about 37% [4], and 5-year overall survival is about 30% [5]. Therefore, the poor prognoses of HCC patients highlight the need for effective adjuvant or postoperative treatments that will improve patients’ long-term outcomes [6].

In recent decades, many types of adjuvant or postoperative treatments for patients with HCC after surgery have been reported [7,8,9,10,11]. However, no adjuvant or postoperative treatments with definite efficacy is found by previous systematic reviews [12,13] or recommended by official guidelines [3,14]. More systematic review with strict inclusion criteria and comprehensive searching is needed.

It is recognized that immnunosuppression induced by surgery is associated with tumor recurrence [15–17]. Therefore, immunotherapy may inhibit growth of HCC cell [18] or even prevent the recurrence of HCC after surgery [19]. In 2012, two systematic reviews [20,21] concluded that adjuvant adoptive immunotherapy (AIT) for patients with HCC after primary treatments may not improve overall survival. Since then, additional studies [22–25] have been published with inconsistent findings. Therefore the present review intends to perform a timely meta-analysis to gain a comprehensive understanding of the available evidence on the benefits and harms of adjuvant AIT for patients with HCC after surgery.

## Methods

### Literature search strategy

A systematic search of PubMed, EMBASE, Scopus and Cochrane Library databases was performed for articles published up to 5 January 2017 relevant to adjuvant AIT for HCC after initially treatment. The systematic review was carried out in accordance with the PRISMA statement for reporting systematic reviews of studies that evaluate healthcare interventions [26]. The following search terms were used to identify comparative studies: ‘hepatocellular carcinoma’ *or* HCC *or* ‘hepatic cancer’ *or* ‘hepatic tumor’ *or* ‘liver tumor’ *or* ‘liver cancer’, *and* ‘hepatic resection’ *or* hepatectomy *or* ‘liver resection’ *or* ‘radiofrequency ablation’ *or* ‘percutaneous ethanol infection’, *and* ‘adoptive immunotherapy’ *or* ‘interleukin-2’ *or* ‘cytokine induced killer cells’ *or* ‘lymphokine activated killer cells’ *or* ‘tumor infiltrating lymphocytes’. Results from the four electronic databases were compared to obtain a list of unique articles for screening. Relevant references of all included studies were also searched manually to identify additional studies. Gray literature was not included in the present analysis.

The following search strategy was used in PubMed (Medline):

(‘hepatocellular carcinoma’ OR HCC OR ‘hepatic cancer’ OR ‘hepatic tumor’ OR ‘liver tumor’ OR ‘liver cancer’) AND (‘hepatic resection’ OR hepatectomy OR ‘liver resection’ OR ‘radiofrequency ablation’ OR ‘percutaneous ethanol infection’) AND (‘adoptive immunotherapy’ OR ‘interleukin-2’ OR ‘cytokine induced killer cells’ OR ‘lymphokine activated killer cells’ OR ‘tumor infiltrating lymphocytes’)

### Selection criteria

The following criteria were applied when considering studies for this meta-analysis:

#### Types of studies

The meta-analysis considered studies evaluating the effectiveness or efficacy of adjuvant AIT for patients with HCC after initial curative treatments. The studies must compare the intervention with no intervention or with a control intervention. Randomized trials, non-randomized trials, and observational studies would be eligible for inclusion.

#### Types of participants

Patients of primary HCC after curative hepatic resection, radiofrequency ablation, percutaneous ethanol injection, or liver transplantation would be included.

#### Types of interventions

Patients in the treatment group received adjuvant AIT. Patients in the control group were without adjuvant AIT.

#### Types of outcome measures

Results must include quantitative data for outcomes measured. The primary outcomes were recurrence rate and mortality. The secondary outcome was treatment-related adverse events, which included treatment-related withdrawals and discontinuations.

Studies were excluded if they evaluated the efficacy of AIT for patients with with recurrent, advanced, or unresectable HCC. Patients who underwent initial TACE were excluded. Conference abstracts and other forms of summary publication were also excluded. In the case of multiple studies apparently based on the same population, only the study with the largest number of participants would be included.

### Data collection

References will be managed using Thomson ISI ResearchSoft EndNote X3 software (Thomson Reuters, New York, USA). Two authors (H.-Y.M, Y.-Y.L) independently screened studies identified in literature searches. Discrepancies were arbitrated by a third author (J.-H.Z). Two authors (H.-Y.M, Y.-Y.L) independently extracted data from included studies using a predefined template. A third author (J.-H.Z) checked the extracted data against the original studies. Survival data were taken directly from tables or the text whenever possible; if such data were presented only in graphs, they were extracted by manual interpolation [27]. *P* values associated with inter-group differences in progression-free survival, disease-free survival, or overall survival were extracted directly from survival curves or text wherever possible.

### Assessment of methodological quality in included studies

For randomized trials, two review authors (H.-Y.M, and Y.-Y.L) independently assessed methodological quality in included studies by considering the following characteristics using the Jadad scale [28]:

- Was the study described as randomized?
- Was the method of randomization described and appropriate?
- Was the study described as double blind?
- Was the method of double blinding described and appropriate?
- Were withdrawals and dropouts described?

For observational and non-randomized trials, two review authors (H.-Y.M, and Y.-Y.L) independently assessed methodological quality using the Newcastle-Ottawa scale [29]. Included case control studies will be assessed by considering the following characteristics:

- Selection of study groups: is the case definition adequate? Are the cases representative? From where are controls selected? Are controls adequately defined?
- Comparability of groups: are cases and controls comparable on the basis of the design or analysis? Ascertainment of exposure/outcome: how is the exposure ascertained? Is the same method of ascertainment of exposure used for cases and controls? Is the non-response rate the same for cases and controls?

Included cohort studies were assessed by considering the following characteristics:

- Selection: is the exposed cohort representative of the general population? Is the non-exposed cohort drawn from the same community as the exposed cohort? How is the exposure ascertained? Is it demonstrated that the outcome of interest was not present at the start of the study?
- Comparability: are the cohorts comparable on the basis of the design or analysis?
- Outcome: how is the outcome assessed? Was the follow-up long enough for outcomes to occur? Was the follow-up of cohorts adequate?

Disagreements between the review authors (H.-Y.M, and Y.-Y.L) over the risk of bias in particular studies would be resolved by discussion, with involvement of a third review author (J.-H.Z) where necessary.

### Data synthesis and analysis

Statistical analysis was performed using Review Manager (RevMan, version 5.3, the Nordic Cochrane Centre, the Cochrane Collaboration, Copenhagen). Pooled risk ratios (RRs) using the Mantel-Haenszel method were calculated for dichotomous data. The Cochrane's Q-statistic and I^2^ index were used to assess statistical heterogeneity in the meta-analysis. For heterogeneous data, a random-effects model was used; otherwise, a fixed-effects model was employed. Publication bias was assessed by visual inspection of Begg’s funnel plots. Sensitivity analyses excluding cohort studies and choice of random- or fixed-effect meta-analysis model were performed. To assess attrition bias, we calculated recurrence and mortality using a ‘worst-case’ approach in which patients with missing data were counted as treatment failures (recurrence or death). For patients with missing data, we 'carried forward' data from the most recent measurement. *P* < 0.05 was considered statistically significant.

## Results

### Study selection

**Fig 1:**
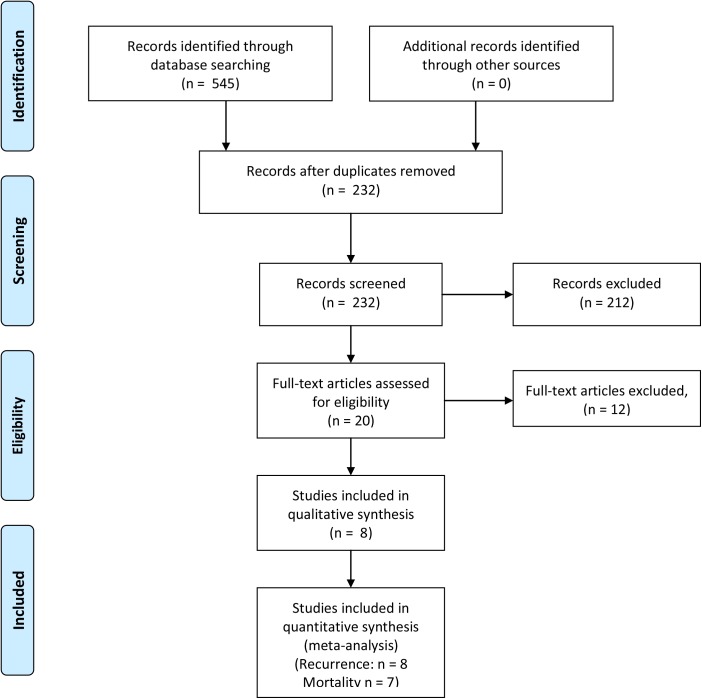
Flow chart of study selection.

A total of 545 potentially eligible studies were identified and reviewed. According to inclusion criteria, 232 studies remained after removing the duplicates. Screening the titles and abstracts led to a final set of 20 studies that were read in full [22–25,30–45]. Of these, six studies [30–35] were excluded because they contained subsets of patients already contained in other larger studies. Three studies investigating AIT for patients with advanced HCC were excluded [36–38], and another study investigating a different type of postoperative immunotherapy was excluded [39]. Two studies including patients who underwent transarterial chemoembolization were also excluded [24,45]. In the end, 7 RCTs [22,23,40–44] and one cohort studies [25] involving 949 AIT-treated and 912 untreated patients were included in the meta-analysis (Fig 1, Table 1).

**Table 1:** Baseline characteristics of included studies.

Study	Study design	Country	Surgery method	Recruitment period	Sample size (T/C)	Child-Pugh score A/B, n	Cirrhosis, n	HBV/HCV, n	Follow-up	Risk of bias
Dong et al. 2009 [40]	randomized	China	Curative resection	2000–2002	84/43	102/25	101	96/-	>5 yr	Low risk
Kawata et al. 1995 [41]	randomized	Korea	Curative resection + adriamycin	1989–1990	12/12	-	14	-	-	High risk
Lee, et al. 2015 [22]	randomized	China	Curative resection, RFA, or PEI	2008–2012	114/112	226/0	146	192/23	About 3 yr	Low risk
Pan et al. 2015 [25]	Retrospective	Japan	Curative resection	2001–2009	511/520	-	-	866/-	-	Low risk
Takayama et al. 2000 [42]	randomized	China	Curative resection	1992–1995	76/74	104/46	73	29/99	Median, 4.4 yr (range, 0.2–6.7)	Low risk
Xie et al. 2000 [43]	randomized	China	Curative resection + TACE	1994–1996	21/21	-	-	-	-	High risk
Xu et al. 2016 [23]	randomized	China	Curative resection	2008–2013	100/100	200/0	113	171/-	Median, 3.2 (range, 0.3–6.1) years	Low risk
Zhou et al. 1995 [44]	randomized	China	Curative resection	1992–1992	31/30	-	-	-/-	-	High risk

*Abbreviations*: -, not reported; HBV, hepatitis B virus; HCV, hepatitis C virus; PEI, percutaneous ethanol injection; RFA, radiofrequency ablation; TACE, transarterial chemoembolization.

### Studies characteristics

All studies came from Asia. One trial [40] contained two AIT-treated arms, one treated with 3 cycles and the other with 6 cycles. Data from the two arms were combined. Patients in one trial also undergoing postoperative transarterial adriamycin chemotherapy [41], and patients in another also receiving postoperative transarterial chemoembolization [43]. Of all patients in the trial by Zhou *et al*. [44], only those who underwent resection alone or resection followed by adjuvant AIT were included in the present meta-analysis; this trial reported recurrence data out to 1 year only [44]. In the trial by Lee and coworkers, most patients underwent hepatic resection, while some of them underwent radiofrequency ablation or percutaneous ethanol injection (Table 1).

### Efficacy

**Fig 2:**
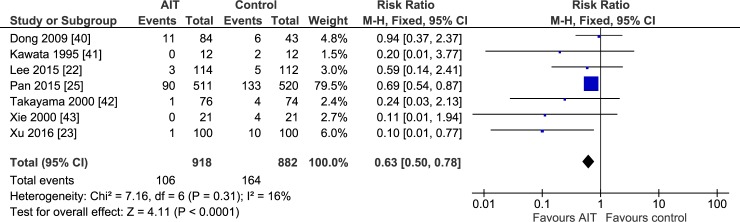
One year recurrence rate of meta-analysis comparing the efficacy of adjuvant adoptive immunotherapy (AIT) with curative treatment alone.

Meta-analysis of 7 studies [22,23,25,40–43] suggested that adjuvant AIT was associated with significantly lower mortality than curative therapies alone at 1 year (Fig 2), 2 years, 3 years, and 5 years (all *P* < 0.05; Table 2). Similar results were obtained using a random- or fixed-effects meta-analysis model. Sensitivity analysis using data from only the 6 RCTs supported a benefit of adjuvant AIT for mortality at 1 year (RR 0.39, 95%CI 0.21–0.72) and 2 years (RR 0.51, 95%CI 0.34–0.76), 3 years (RR 0.71, 95%CI 0.55–0.92), but not at 5 years (RR 0.99, 95%CI 0.83–1.19).

**Table 2:** Results of meta-analyses.

Outcomes	No. of studies	Heterogeneity of study design			Fixed-effect model		Random-effect model	
		χ^2^	df (P value)	I^2^	Risk ratio [95% CI]	Z (P value)	Risk ratio [95% CI]	Z (P value)
Mortality								
1 year	7	7.16	6 (0.31)	16	0.63 [0.50, 0.78]	4.11 (<0.001)	0.59 [0.38, 0.92]	2.34 (0.02)
2 years	7	6.50	6 (0.37)	0	0.72 [0.63, 0.83]	4.44 (<0.001)	0.70 [0.56, 0.87]	3.20 (0.001)
3 years	7	3.83	6 (0.70)	0	0.74 [0.65, 0.83]	5.03 (<0.001)	0.74 [0.66, 0.83]	5.02 (<0.001)
5 years	3	2.14	2 (0.34)	6	0.88 [0.80, 0.96]	2.79 (0.005)	0.89 [0.80, 0.98]	2.36 (0.02)
Tumor recurrence								
1 year	8	15.30	7 (0.03)	54	0.80 [0.71, 0.90]	3.79 (0.001)	0.65 [0.47, 0.89]	2.68 (0.007)
2 years	7	16.52	6 (0.01)	64	0.80 [0.73, 0.88]	4.84 (<0.001)	0.68 [0.54, 0.86]	3.20 (0.001)
3 years	7	3.83	6 (0.70)	0	0.86 [0.80, 0.93]	3.91 (<0.001)	0.86 [0.80, 0.93]	3.97 (<0.001)
5 years	3	0.37	2 (0.83)	0	0.91 [0.85, 0.97]	2.90 (0.004)	0.91 [0.85, 0.97]	2.95 (0.003)

**Fig 3:**
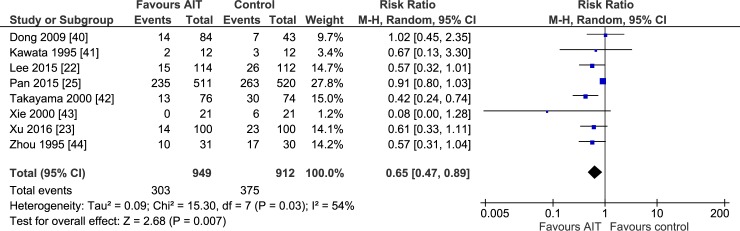
One year mortality of meta-analysis comparing the efficacy of adjuvant adoptive immunotherapy (AIT) with curative treatment alone.

Meta-analysis of all 8 studies [22,23,25,40–44] suggested that adjuvant AIT was associated with significantly lower recurrence rate than curative therapies alone at 1 year (Fig 3), 2 years, 3 years, and 5 years (Table 2). Similar results were obtained using a random- or fixed-effects meta-analysis model. In addition, intermediate statistically significance was observed in 1 and 2 years. After excluding the non-randomized study [25], results confirmed the recurrence benefit of adjuvant AIT at 1 year (RR 0.57, 95%CI 0.43–0.74), 2 years (RR 0.63, 95%CI 0.52–0.76) and 3 years (RR 0.81, 95%CI 0.71–0.93) (all *P* < 0.05). However, adjuvant AIT did not significantly reduce 5-year recurrence rate in this sensitivity analysis (RR 0.92, 95%CI 0.83–1.02). In addition, no statistically significance was observed after excluding the non-randomized study in any of the meta-analyses.

### Safety

No hospital deaths or serious adverse events attributed to adjuvant AIT was reported in the 8 studies. The most frequent adverse events were grade 1 fever (defined as persistent or transient temperature of 37.5–39.3°C) and chills. In patients experiencing fever, the condition was easily controlled with symptomatic therapies. Rare adverse events included nausea, headache, fatigue, myalgia, dizziness, itching, and tachycardia. All adverse events were grade 1 or 2 and self-limiting.

### Risk of bias of included studies

The Jadad Scale [28] was used to assess risk of bias for randomized trials (Table 3). Four RCTs [22,23,40,42] had a high methodological quality score (≥3) and three RCTs [41,43,44] had a low methodological quality score (≤2). Owing to the difficulties in blinding the intervention, none of the RCTs were able to score the maximum of five points; therefore a score of three, which is classified as high methodological quality, was the maximum score possible. Risk of bias of the non-randomized study was assessed by Newcastle-Ottawa scale [29]. This study was judged to have low risk of bias [25].

**Table 3:** Jadad Scale representing scores in descending order.

Study	Described as RCT?	Adequate randomization?	Double-blind?	Details of double-blinding?	Reasons stated for withdrawals?	Total score
Dong et al. 2009 [40]	+1	+1	+0	+0	+1	3
Kawata et al. 1995 [41]	+0	+0	+0	+0	+0	0
Lee, et al. 2015 [22]	+1	+1	+0	+0	+1	3
Takayama et al. 2000 [42]	+1	+1	+0	+0	+1	3
Xie et al. 2000 [43]	+1	+0	+0	+0	+0	1
Xu et al. 2016 [23]	+1	+1	+0	+0	+1	3
Zhou et al. 1995 [44]	+1	+0	+0	+0	+0	1

### Assessment of publication bias

Funnel plots of the 8 studies in the 1 year mortality or recurrence rate meta-analyses showed a symmetrical shape, suggesting minimal risk of publication bias.

## Discussion

This timely meta-analysis found adjuvant AIT was safe for patients with primary HCC after curative hepatic resection, radiofrequency ablation, or percutaneous ethanol injection. Moreover, in contrast to those previous systematic reviews [20,21], our data found adjuvant AIT significantly reduced the rate of mortality and tumor recurrence for such patients. Our results were confirmed by other meta-analysis that AIT was a feasible adjuvant treatment for the improvement of the clinical outcomes for HCC patients after minimally invasive treatment [46].

Even after curative treatment, HCC is associated with a high recurrence rate. Moreover, recurrence is the primary cause of death of all patients with HCC. Previous meta-analysis found adjuvant transarterial chemoembolization shows promise for reducing recurrence and mortality for HCC patients with high risk of recurrence [47]. In addition, postoperative antiviral therapy with nucleoside/nucleotide analogues can be safe and effective treatment for patients with hepatitis B virus-related HCC [48,49]. However, some HCC patients are unfit for adjuvant transarterial chemoembolization or postoperative antiviral therapy after surgery. For these patients, and for those with low immune function, which is associated with HCC recurrence [50], adjuvant AIT may prevent tumor relapse. Adjuvant AIT involves transferring immune effectors into the cancer patient in the hopes of stimulating specific anti-tumor immune responses [51]. Such stimulation may counterbalance the strongly immunosuppressive microenvironment in the liver [52].

The discrepancy between our findings and those of previous systematic reviews likely reflects the more than two decades spanned by the literature, with the first randomized trial on adjuvant AIT for HCC after surgery published in 1995 [41,44] and the most recent in 2016 [23], combined with rapid scientific and technological advances in AIT [53,54]. In addition, no international guidelines or standards exist regarding route of AIT administration, dosing, or cycles. As a result, clinicians can vary substantially in what immune effector cells they use for AIT and what dosing/cycling protocols they follow. Indeed, in this timely meta-analysis, AIT was based on three types of immunological effector cells: anti-CD3–activated peripheral blood lymphocytes, cytokine-induced killer cells, and lymphokine-activated killer cells. AIT was administered via injection into the intrahepatic artery [43,44] or via intravenous infusion [22,23,25,42]. The number of cycles varied from one [43] to 16 [22]. Such heterogeneity highlights the importance of evidence updates like the present one, and the need for systematic assessment and optimization of AIT protocols, perhaps even tailored to HCC type or treatment history.

Our meta-analysis of two randomized data [40,42] suggests that adjuvant AIT did not significantly reduce mortality and recurrence rate at 5 years. This may mean that AIT-mediated immune boosting can eliminate small intrahepatic metastases, but it does not prevent multicentric relapse in remnant liver. This hypothesis is consistent with the findings of one study [23] that the ability of adjuvant AIT to prevent tumor recurrence was more obvious in the short term and less so in the long term, and that its ability to prolong time to recurrence was greater in patients with tumors >5 cm, moderately or poorly differentiated tumors, or preoperative α-fetoprotein levels ≥25 ng/mL. Adjuvant AIT may have no effect on liver cirrhosis of remnant liver, which is the main risk factor of postoperative recurrence of de novo HCC [55,56]. The effects of adjuvant AIT on HCC recurrence in the short and long term should be investigated in greater detail.

Our timely meta-analysis of the safety and efficacy of adjuvant AIT for patients with primary HCC after curative therapies rests on four randomized trials with a low risk of bias and other studies with a high risk bias and is dominated by Asian populations. Similar results may not be observed in Western populations. Second, type of cytokines, number of infusion cycles, and duration of maintenance AIT therapy varied among the included studies, creating substantial heterogeneity for which we could not control using sensitivity analyses. In addition, length of follow-up varied across the studies and in some cases was too short to observe long-term efficacy of adjuvant AIT. As a result, meta-analysis of outcomes at 3 and 5 years had to be conducted on subsets of all included studies. Some studies did not clearly report procedures for randomization or allocation concealment, increasing the risk of selection or reporting bias. The findings must be interpreted with caution.

## Conclusion

Our timely meta-analysis provides an updated picture of the evidence based on adjuvant AIT: AIT is safe and effective in reducing mortality and tumor recurrence for patients with HCC after curative therapies. Regarding future research, a randomized trial with adequate follow-up is highly desirable. In addition, this study should aim to expand the range of relevant endpoints examined, such as quality of life, duration of hospital stay, and cost-effectiveness. And last but not least, this study should also examine the possible clinical benefits of multi-modal immune therapies.

## Supporting information

S1 PRISMA Checklist PRISMA 2009 checklist. (DOC)
